# Extending TextAE for annotation of non-contiguous entities

**DOI:** 10.5808/GI.2020.18.2.e15

**Published:** 2020-06-15

**Authors:** Jake Lever, Russ Altman, Jin-Dong Kim

**Affiliations:** 1 Department of Bioengineering, Stanford University, Stanford, CA 94305, USA; 2 Database Center for Life Science, Research Organization of Information and Systems, Kashiwa 277-0871, Japan

**Keywords:** editor, text annotation, text mining, visualization

## Abstract

Named entity recognition tools are used to identify mentions of biomedical entities in free text and are essential components of high-quality information retrieval and extraction systems. Without good entity recognition, methods will mislabel searched text and will miss important information or identify spurious text that will frustrate users. Most tools do not capture non-contiguous entities which are separate spans of text that together refer to an entity, e.g., the entity “type 1 diabetes” in the phrase “type 1 and type 2 diabetes.” This type is commonly found in biomedical texts, especially in lists, where multiple biomedical entities are named in shortened form to avoid repeating words. Most text annotation systems, that enable users to view and edit entity annotations, do not support non-contiguous entities. Therefore, experts cannot even visualize non-contiguous entities, let alone annotate them to build valuable datasets for machine learning methods. To combat this problem and as part of the BLAH6 hackathon, we extended the TextAE platform to allow visualization and annotation of non-contiguous entities. This enables users to add new subspans to existing entities by selecting additional text. We integrate this new functionality with TextAE’s existing editing functionality to allow easy changes to entity annotation and editing of relation annotations involving non-contiguous entities, with importing and exporting to the PubAnnotation format. Finally, we roughly quantify the problem across the entire accessible biomedical literature to highlight that there are a substantial number of non-contiguous entities that appear in lists that would be missed by most text mining systems.

## Introduction

Information extraction and retrieval methods are essential tools to enable scientists to find and read the appropriate papers to enable discoveries. Many of these methods require identifying mentions of specific biomedical entities in the text and make use of named entity recognition (NER) tools for this task. Most entities are represented by a single span of text, e.g., the name of a drug. However, some entities are represented by multiple spans of text that are separated by other words and together identify the entity, for example, the separate words “skin” and “cancer” in “skin and lung cancer.” These are known as non-contiguous, or discontiguous entities. Table 1 illustrates several more examples from public text mining resources. It should be noted that non-contiguous entities are different from anaphora or coreference resolution, in which multiple spans refer to the same entity separately and do not work together to identify the entity.

Robust annotation tools that are capable of annotating non-contiguous entities are important so that valuable entity information is not missed. These tools are needed to create corpora with non-contiguous entities that can be used as training data for machine learning-based NER methods and also evaluate all NER methods fairly. The leading NER methods frequently use machine-learning methods such as conditional random fields (CRF) that are incapable of capturing non-contiguous entities without additional postprocessing. Popular tools such as BANNER [1], tmChem [2], and DNorm [3] do not support non-contiguous entities.

Many annotation tools have been developed for manual tagging of entities within a document for the biological domain and other domains. A detailed recent review of the strengths and weaknesses of different methods can be found in Neves and Seva’s study [4]. To gauge the support for non-contiguous entities, we manually tested the 15 tools selected in that review with an overview shown in Table 2. We were able to run all but one, PDFAnno which displayed an error message that others have reported on Github. We found that only 2 support non-contiguous entities, BRAT [5], and Catma. Furthermore the AlvisAE [6] tool that was not included in the review also supports non-contiguous entity annotation. We suggest that more tools need to provide support for non-contiguous entities.

**Fig. 1.**
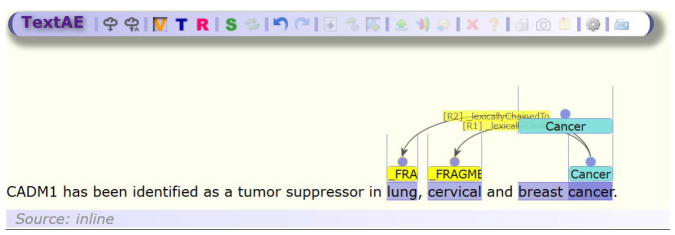
Illustration of three entity annotations including two non-contiguous represented using the older chaining model which is cumbersome to annotate and visually cluttered.

To that goal, we describe the addition of non-contiguous entity support to TextAE. TextAE is an annotation platform that forms part of the PubAnnotation system for storing and editing text annotations [7]. It is a Node.js web component that accepts text annotations in PubAnnotation JSON format. The PubAnnotation format currently has support for non-contiguous entities but are converted to an alternative representation when edited using the current release of TextAE, known as the chaining representation. This representation converts an entity that contains multiple spans to multiple entities and links them with a relation with type “_lexicallyChainedTo.” This representation is time-consuming to edit and visualizes poorly. Fig. 1 illustrates the current representation of three non-contiguous entities within a sentence using the chaining method. With the current TextAE interface, it is time-consuming to annotate each entity. Assuming TextAE has been set up with appropriate entity and relation types, for each non-contiguous entity, it requires creating two entities (2 mouse clicks), designating one entity with the type “_FRAGMENT” (2 clicks), switching to the relation mode (1 click), creating a relation between the two entities (2 clicks), changing its type to “_lexicallyChainedTo” (2 clicks) and switching back to Term Mode to continue entity annotation (1 click). Even for a TextAE power user, ten clicks for each non-contiguous entity is time-consuming for a large-scale annotation and produces an unwieldy result which is not visually clear.

In this paper, we describe our solution of an extension to the existing TextAE annotation platform to provide seamless support for annotating non-contiguous entities. Finally, we provide evidence of the widespread nature of non-contiguous entities in the biomedical literature using a rule-based extraction system to roughly quantify the scale of non-contiguous entities across all PubMed abstracts and accessible PubMed Central full-text papers.

## Methods

**Fig. 2.**
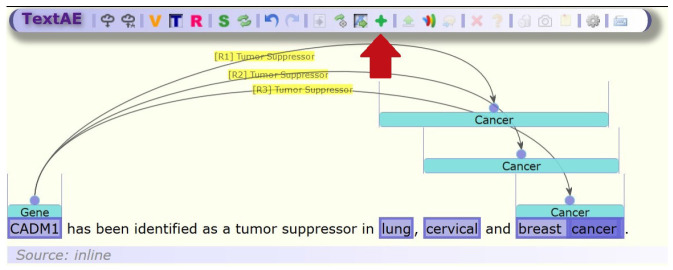
The user interface with the new Add subspan button in the toolbar (highlighted by red arrow) and non-contiguous entities annotated as part of three relationships.

To develop improved methods to capture non-contiguous entities, well-annotated data needs to be prepared and examined that contain non-contiguous entities. We extend the TextAE annotation platform that is part of the PubAnnotation system [7]. This enables annotation of entities with multiple spans as shown in Fig. 2 with a new subspan mode. The user can select new spans of text that will be added to an existing entity and displayed clearly.

The first task for implementation was changing the underlying span model in TextAE so that all spans are represented as a list of subspans. We dynamically check the input annotation data (in PubAnnotation format) to check if an entity has a single span, or a list of spans, and convert all entities to contain lists of spans, even for single spans. Previously, spans were rendered using a single HTML span tag around the section with appropriate CSS styling to identify the span as an entity. To visualize the new subspans, we removed the styling from the span class, and create subspans for each part of the span and transferred the stylings to the subspans. TextAE implements an Undo/Redo system so changes were required across the codebase to enable the existing functionality to work with the new underlying data structure and allow entities to be manipulated as before.

A new toggleable button (Add subspan) was added to the toolbar. When this button is toggled, any new spans that are selected by the user are added to the previously selected entity. This requires checking that new subspans were compatible with the structure that is enforced by the HTML page. This means that spans and subspans cannot intersect unless one is contained within the other entirely. This means that in the snippet: “breast cancer gene”, it would not be possible for “breast cancer” and “cancer gene” to be tagged as entities. However “breast cancer” and “cancer” could be tagged as “cancer” is fully contained within “breast cancer.” We have not come across use-cases where this functionality is currently needed but cannot discount the potential of this limitation. Fig. 2 illustrates the user interface with an example of non-contiguous entities.

TextAE has several user interface shortcuts to enable fast annotation and correction of entities. Users can extend an entity annotation by highlighting text that begins within an entity annotation and goes beyond the entity. Inversely, users can also shorten entity annotations by highlighting text that begins outside an entity span and finishes within an entity, thereby removing the selected text from the entity annotation. We extended this functionality to work for the new subspan system so that it would extend the appropriate first or last subspan in an entity outwards, or would shorten or even remove subspans that are highlighted. We further added user interface tweaks so that when a user selected a subspan, it would select all the subspans for the entity. Finally, we implemented export functionality so that the new subspans would be correctly stored in the PubAnnotation format with a list of spans for those entities with multiple subspans.

The code for this paper is available at https://github.com/jakelever/textae.

## Results

We first tested to check that all existing functionality of TextAE remained operational. We confirmed that the new subspan model was able to load data containing non-contiguous entities and annotations with non-contiguous entities could be saved correctly to the PubAnnotation format. Furthermore, we tested that all existing functionality, including relation annotation, worked correctly with non-contiguous entity annotations.

We quantified the user interactions required to annotate non-contiguous entities. With this new interface, the user needs to annotate a single span (1 click), enable the Add subspan mode (1 click), add a new subspan (1 click), and disable the Add subspan mode (1 click). With only four clicks, we have drastically reduced the user effort, compared to the 10 clicks required previously, and no longer require the user to switch annotation modes within TextAE. Furthermore, the output is visually clearer. This performance is similar to the Catma tool, which requires four clicks to annotate a non-contiguous entity (1 to activate the discontinuous mode, 2 to select the two spans and 1 to select the entity type). And it is marginally easier than BRAT which takes five clicks (2 to annotate the first entity, 1 to edit the entity, 1 to select Add Frag and 1 to select the new span).

## Discussion

While non-contiguous entities initially seem like a limited problem for text annotation, we note that two other BLAH 6 hackathon projects requested this functionality during the event: a project working on annotations from the recent BioNLP Shared Task [10] and a project focused on Medical Device Indication annotation. To understand the scale of this problem, we quantified the number of non-contiguous entities that appear in lists, as shown in the CancerMine examples in Table 1. We focussed on this format as these can be extracted using a modified dictionary matching method.

We used the PubTator Central resource [11] as it provides text-level entity annotations of a very large set of biomedical publications and also a rough set of synonyms for different entity types. The annotations provide locations of biomedical entities that may be the final element in a list. For example, the phrase “prostate, skin and lung cancer” would only likely be tagged for “lung cancer” in PubTator. We aimed to retrieve other entities from these lists using the set of synonyms from PubTator Central, so that “prostate cancer” and “skin cancer” would be extracted from the example phrase. We used a simple rule-based system that identified candidate lists by searching for tagged biomedical entities that follow the word “and.” We then searched the preceding words in the candidate list and attempted word substitutions with the final term to find terms that were in the lexicon.

Across the 30,044,935 abstracts and 2,485,641 full-text papers that were minable, we find 3,269,632 potential mentions of non-contiguous entities in the example list format. We manually reviewed 100 of them to understand the error profile and found that 42% were true positives. The main errors were caused by spurious mistakes in the lexicon and a more conservative lexicon would likely improve precision but may affect overall recall. Nevertheless, this initial result suggests that many biomedical entities are described in the list form that would be missed with most current methods. While there are considerable false-positive dues to the dictionary matching method, we would argue that this will only be a fraction of non-contiguous entities across the biomedical literature as we examine only one type of linguistic structure that could contain non-contiguous entities.

**Fig. 3.**
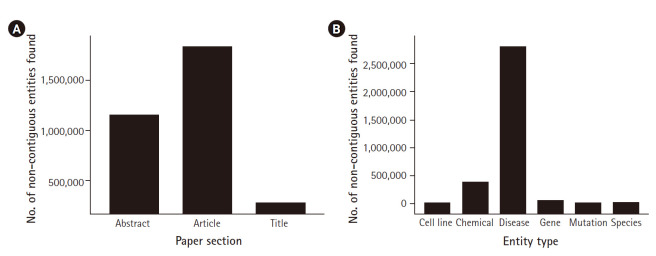
The non-contiguous entities grouped the section of the paper (A) and the entity type (B).

Fig. 3 shows an overview of the results from the literature analysis. Lists appear more in full-text papers than in abstracts even when taking account of the substantially larger number of abstracts than full-text articles in the corpus. They can even appear in the article title. Furthermore, disease has substantially more non-contiguous entities, likely due to the larger number of multiple word terms in that lexicon (837,390 compared to 103,427 for genes for example).

This analysis strongly suggests that non-contiguous are a substantial problem in biomedical text mining and that methods that ignore them will be missing large amounts of potential extracted information. We hope our contribution to an annotation tool that could help visualize and annotate these problematic entities may take a step towards new methods to identify them.

## Figures and Tables

**Table 1.** Examples of non-contiguous entities from different public text mining datasets

Source	PubMed ID	Snippet	Non-contiguous entity
BioNLP 2019 Bacteria Biotope Task	23224222	Both **French** and German **cheeses** have previously been reported to contain M. psychrotolerans	French cheeses
	19622846	...and used API tests to identify S. aureus and E-tests to determine **methicillin**/oxacillin **resistance**	Methicillin resistance
CancerMine	19855840	It is suggested that DLC1 is a candidate tumour suppressor gene for human liver cancer, as well as for **prostate**, lung, colorectal and breast **cancers**	Prostate cancers
	19734946	LARG at chromosome 11q23 has functional characteristics of a tumor suppressor in human **breast **and colorectal **cancer**	Breast cancer
PGxMine	23385314	In vitro analysis and quantitative prediction of efavirenz inhibition of eight cytochrome P450 (CYP) enzymes: major effects on **CYP**s 2B6, 2C8, 2C9 and **2C19**	CYP 2C19

The examples from CancerMine [8] and PGxMine [9] are not currently captured by the corresponding method and are false negatives.

**Table 2.** An analysis of the annotation tools reviewed in Neves and Seva’s study [4] for their capabilities to annotate non-contiguous entities

Tool	URL	Can run?	Supports entity annotation?	Support non-contiguous entities?
BioQRator	http://www.bioqrator.org	Y	Y	N
brat	http://brat.nlplab.org	Y	Y	Y
Catma	https://catma.de	Y	Y	Y
Djangology	https://sourceforge.net/projects/djangology	Y	Y	N
ezTag	https://eztag.bioqrator.org	Y	Y	N
FLAT	https://github.com/proycon/flat	Y	Y	N
LightTag	https://www.lighttag.io	Y	Y	N
MAT	http://mat-annotation.sourceforge.net	Y	Y	N
MyMiner	http://myminer.armi.monash.edu.au	Y	Y	N
PDFAnno	https://github.com/paperai/pdfanno	N	-	-
prodigy	https://prodi.gy/	Y	Y	N
tagtog	https://www.tagtog.net/	Y	Y	N
WAT-SL	https://github.com/webis-de/wat	Y	N	-
WebAnno	https://webanno.github.io	Y	Y	N
